# Lacunary Series and Strong Approximation †

**DOI:** 10.3390/e27020214

**Published:** 2025-02-19

**Authors:** István Berkes

**Affiliations:** Rényi Mathematical Institute, Reáltanoda u. 13-15, 1053 Budapest, Hungary; berkes@renyi.hu

**Keywords:** lacunary series, limit theorems, exchangeability, subsequence principle, conditional independence, 60F05, 60F15, 60G09

## Abstract

Strong approximation, introduced by Strassen (1964), is one of the most powerful methods to prove limit theorems in probability and statistics. In this paper we use strong approximation of lacunary series with conditionally independent sequences to prove uniform and permutation-invariant limit theorems for such series.

## 1. Introduction

It is known that sufficiently thin subsequences of any (dependent) sequence of random variables behave like independent random variables. Révész [1] proved that if ${\left(X_{n}\right)}_{n\geq 1}$ is a sequence of random variables satisfying $\sup_{n}E\left(X_{n}^{2}\right)<\infty$, then there exists a subsequence ${\left(X_{n_{k}}\right)}_{k\geq 1}$ and a random variable $X\in L_{2}$ such that the series $\sum_{k=1}^{\infty }a_{k}\left(X_{n_{k}}-X\right)$ converges a.s. for any coefficient sequence ${\left(a_{k}\right)}_{k\geq 1}$ with $\sum_{k=1}^{\infty }a_{k}^{2}<\infty$. Komlós [2] proved that from any sequence ${\left(X_{n}\right)}_{n\geq 1}$ of random variables satisfying $\sup_{n}E\left|X_{n}\right|<\infty$, one can select a subsequence ${\left(X_{n_{k}}\right)}_{k\geq 1}$ such that

(1) $$\lim_{N\to \infty }\frac{1}{N}\sum_{k\leq N}X_{n_{k}}=X\;a.s.$$

for some $X\in L_{1}$. Gaposhkin [3] and Chatterji [4,5] proved that if ${\left(X_{n}\right)}_{n\geq 1}$ is a sequence of random variables satisfying $\sup_{n}EX_{n}^{2}<\infty$, then there exist a subsequence ${\left(X_{n_{k}}\right)}_{k\geq 1}$ and random variables $X\in L_{2}$, $Y\in L_{1}$, Y≥0 such that

(2) $$\frac{1}{\sqrt{N}}\sum_{k\leq N}\left(X_{n_{k}}-X\right)\overset{d}{⟶}N\left(0,Y\right)$$

and

(3) $$\underset{N\to \infty }{lim sup}\;\frac{1}{\sqrt{2N\log\log N}}\sum_{k\leq N}\left(X_{n_{k}}-X\right)=Y^{1/2}\;a.s.,$$

where N(0,Y) denotes the distribution of $\sqrt{Y}g$; here *g* is an N(0,1) variable independent of *Y*. Chatterji [6] formulated the following heuristic principle:

**Subsequence Principle.**  *Let T be a probability limit theorem valid for all sequences of i.i.d. random variables belonging to an integrability class L defined by the finiteness of a norm ${∥\;\cdot ∥}_{L}$. Then, if ${\left(X_{n}\right)}_{n\geq 1}$ is an arbitrary (dependent) sequence of random variables satisfying $\sup_{n}{∥X_{n}∥}_{L}<\infty$, then there exists a subsequence ${\left(X_{n_{k}}\right)}_{k\geq 1}$ satisfying T in a mixed form.*

In a profound study, Aldous [7] proved the validity of the subsequence principle for all distributional and almost sure limit theorems subject to minor technical conditions. Dacunha-Castelle [8] showed that every tight sequence ${\left(X_{n}\right)}_{n\geq 1}$ has a subsequence ${\left(X_{n_{k}}\right)}_{k\geq 1}$ whose finite dimensional distributions are close to those of an exchangeable sequence ${\left(Y_{k}\right)}_{k\geq 1}$, defined on a possibly different probability space. However, the closeness of the finite dimensional distributions of ${\left(X_{n_{k}}\right)}_{k\geq 1}$ and ${\left(Y_{k}\right)}_{k\geq 1}$ is not enough to transfer limit theorems from ${\left(X_{n_{k}}\right)}_{k\geq 1}$ to ${\left(Y_{k}\right)}_{k\geq 1}$ and Aldous [7] used a delicate subsequence extraction technique tailored to the individual limit theorem we want to establish for $\left(X_{n_{k}}\right)$.

An alternative way to prove Aldous’ theorem would be to use strong approximation and to show that every tight sequence ${\left(X_{n}\right)}_{n\geq 1}$ has a subsequence ${\left(X_{n_{k}}\right)}_{k\geq 1}$ which is close to an exchangeable sequence ${\left(Y_{k}\right)}_{k\geq 1}$ defined on the same probability space in the sense that

(4) $$\sum_{k=1}^{\infty }\left|X_{n_{k}}-Y_{k}\right|<\infty \;a.s.$$

Clearly, by passing to a further subsequence, one can make the speed of a.s. convergence of $X_{n_{k}}-Y_{k}$ to 0 as rapid as one wishes; then, transferring limit theorems from ${\left(Y_{k}\right)}_{k\geq 1}$ to ${\left(X_{n_{k}}\right)}_{k\geq 1}$ becomes easy. Unfortunately, however, the approximation (4) is not valid in general, as is shown by the examples in [7,9]. For a necessary and sufficient condition on $\left(X_{n}\right)$ to have a subsequence ${\left(X_{n_{k}}\right)}_{k\geq 1}$ satisfying (4) with an exchangeable ${\left(Y_{k}\right)}_{k\geq 1}$, see [9]. However, in [10] we showed that a slightly weaker version of (4) is nevertheless true, namely we have

**Theorem** **1.** *Let ${\left(X_{n}\right)}_{n\geq 1}$ be an arbitrary (not necessarily tight) sequence of random variables with tail σ-field T. Then, after suitably enlarging the probability space, there exists a subsequence ${\left(X_{n_{k}}\right)}_{k\geq 1}$ and a sequence ${\left(Y_{k}\right)}_{k\geq 1}$ of random variables such that $Y_{k}$ are conditionally independent with respect to T and (4) holds.*

Theorem 1 is an almost sure invariance principle in the sense of Strassen [11], but it yields a.s. approximation of the individual r.v’s $X_{n_{k}}$, instead of their partial sums.

By De Finetti’s theorem, the exchangeability of a sequence ${\left(Y_{k}\right)}_{k\geq 1}$ can be “split” into two properties, namely, to the conditional independence of ${\left(Y_{k}\right)}_{k\geq 1}$ relative to its tail σ-field T and the conditional identical distribution of ${\left(Y_{k}\right)}_{k\geq 1}$ relative to T. As Theorem 1 shows, the conditional independence of ${\left(Y_{k}\right)}_{k\geq 1}$ can always be guaranteed in (4) and the difficulties are caused by the second, seemingly much simpler property, the conditional identical distribution of the $Y_{k}$’s. In [10] we gave an example showing that even in the case when the sequence ${\left(X_{n}\right)}_{n\geq 1}$ is uniformly bounded, the behavior of the conditional distribution functions $F_{k}\left(t\right)=P\left(Y_{k}<t|T\right)$ of $Y_{k}$ can be extremely irregular, with no subsequence $F_{m_{k}}$ converging in any useful sense. However, even without the conditional identical distribution of the $Y_{k}$’s, the approximation (4) has many useful consequences. As the examples in the next section will show, Theorem 1 provides important information for nonstationary lacunary series, e.g., for the a.s. convergence and asymptotic properties of series $\sum a_{k}X_{n_{k}}$. For example, while the Aldous–Chatterji subsequence theory yields the Hartman–Wintner-type LIL (3) for sufficiently thin subsequences ${\left(X_{n_{k}}\right)}_{k\geq 1}$ of $L_{2}$-bounded sequences ${\left(X_{n}\right)}_{n\geq 1}$, Theorem 1 yields the Kolmogorov-type LIL

(5) $$\underset{N\to \infty }{lim sup}\frac{\sum_{k=1}^{N}a_{k}\left(X_{n_{k}}-X\right)}{\sqrt{2A_{N}^{2}\log\log A_{N}}}=Y^{1/2}\;\;a.s.$$

for suitable subsequences ${\left(X_{n_{k}}\right)}_{k\geq 1}$ of uniformly bounded sequences ${\left(X_{n}\right)}_{n\geq 1}$, where ${\left(a_{n}\right)}_{n\geq 1}$ is a weight sequence satisfying

(6) $$A_{N}={\left(\sum_{k=1}^{N}a_{k}^{2}\right)}^{1/2}⟶\infty ,\;\;\max_{k\leq N}\left|a_{k}\right|=o\left(\frac{A_{N}}{\sqrt{\log\log A_{n}}}\right).$$

In conclusion we note that in [12] it is proved that for any tight sequence ${\left(X_{n}\right)}_{n\geq 1}$, there is a subsequence ${\left(X_{n_{k}}\right)}_{k\geq 1}$ and a sequence ${\left(Y_{k}\right)}_{k\geq 1}$ of random variables which is ‘strongly exchangeable at infinity’ in a certain technical sense and (4) holds. Thus, we have an a.s. approximation theorem even in the context of the Chatterji–Aldous subsequence theory, but it applies only for limit theorems for i.i.d. or near-i.i.d. sequences.

## 2. Applications

By the result of Révész [1] cited in the Introduction, if ${\left(X_{n}\right)}_{n\geq 1}$ is a sequence of r.v.’s with $\sup_{n}E\left(X_{n}^{2}\right)<\infty$, then there exist a subsequence ${\left(X_{n_{k}}\right)}_{k\geq 1}$ and a random variable $X\in L_{2}$ such that ${\left(X_{n_{k}}-X\right)}_{k\geq 1}$ is a convergence system, i.e., for any numerical sequence ${\left(c_{k}\right)}_{k\geq 1}$ with $\sum_{k=1}^{\infty }c_{k}^{2}<\infty$, the series $\sum_{k=1}^{\infty }c_{k}\left(X_{n_{k}}-X\right)$ converges almost surely. Komlós [13] proved that with a suitable choice of $\left(n_{k}\right)$ and *X*, ${\left(X_{n_{k}}-X\right)}_{k\geq 1}$ will actually be an unconditional convergence system (i.e., it will be a convergence system after any permutation of its terms), settling a long-standing open problem in the theory of orthogonal series, see [14], p. 54. Another, equally elaborate proof of Komlós’ theorem was given by Aldous [7]. Since conditional independence is a permutation-invariant property, the two-series version of the Kolmogorov three-series criterion and Beppo Levi’s theorem imply that a conditionally independent sequence with conditional mean 0 and bounded second moments is an unconditional convergence system. Thus, observing that (4) and $\sum_{k=1}^{\infty }c_{k}^{2}<\infty$ imply

$$\sum_{k=1}^{\infty }|c_{k}\left|\right|X_{n_{k}}-Y_{k}|\leq {\left(\sum_{k=1}^{\infty }c_{k}^{2}\right)}^{1/2}{\left(\sum_{k=1}^{\infty }{\left|X_{n_{k}}-Y_{k}\right|}^{2}\right)}^{1/2}<\infty \;\;a.s.,$$

the following version of Komlós’ theorem follows immediately from Theorem 1:

**Theorem** **2.** *Let ${\left(X_{n}\right)}_{n\geq 1}$ be a sequence of r.v.’s with tail σ-algebra T such that $\sup_{n}E\left(X_{n}^{2}\right)<\infty$. Then ${\left(X_{n}-E\left(X_{n}|T\right)\right)}_{n\geq 1}$ contains an unconditional convergence system.*

By passing to a further subsequence and using a weak compactness theorem, the centering sequence $E(X_{n}|T)$ can be replaced by a single random variable $X\in L_{2}$.

Proving the unconditionality of a convergence system is a difficult problem of analysis, with a number of famous results and open problems. By Carleson’s theorem, ${\left(\cos nx\right)}_{n\geq 1}$ is a convergence system, but, as Kolmogorov pointed out in [15], this property breaks down after a suitable permutation of the sequence. In the opposite direction, Garsia [16] showed that if ${\left(f_{n}\right)}_{n\geq 1}$ is an orthonormal system and $\sum_{n=1}^{\infty }c_{n}^{2}<\infty$, then the sum $\sum_{n=1}^{\infty }c_{n}f_{n}$ is a.e. convergent after a suitable permutation of its terms. But whether there is a universal permutation σ:N→N of the positive integers such that $\sum_{n=1}^{\infty }c_{n}f_{\sigma (n)}$ is a.e. convergent for any coefficient sequence ${\left(c_{n}\right)}_{n\geq 1}$ with $\sum_{n=1}^{\infty }c_{n}^{2}<\infty$ (i.e., ${\left(f_{\sigma (n)}\right)}_{n\geq 1}$ is a convergence system) is still an open question.

As mentioned above, the subsequence ${\left(n_{k}\right)}_{k\geq 1}$ constructed in the proof of Aldous’ theorem depends on the limit theorem we want to verify and different limit theorems require different subsequences. For example, Aldous’ theorem implies that if $\left(X_{n}\right)$ is a sequence of r.v.’s with $\sup_{n}E\left(X_{n}^{2}\right)<\infty$, then for any fixed numerical sequence ${\left(c_{k}\right)}_{k\geq 1}$ with $\sum_{k=1}^{\infty }c_{k}^{2}<\infty$, there exist a subsequence ${\left(X_{n_{k}}\right)}_{k\geq 1}$ and $X\in L_{2}$ such that $\sum_{k=1}^{\infty }c_{k}\left(X_{n_{k}}-X\right)$ converges a.e., but the theorem does not yield a universal subsequence ${\left(X_{n_{k}}\right)}_{k\geq 1}$ working for all square summable sequences ${\left(c_{k}\right)}_{k\geq 1}$ simultaneously, and thus Révész’ theorem mentioned in the Introduction does not follow from Aldous’ theorem. The same applies for the weighted CLT and LIL for lacunary sequences proved by Gaposhkin (see [17], Theorem 1.5.1 and Theorem 1.6.1). However, both results are immediate consequences of Theorem 1 and since conditional independence is a permutation-invariant property, we automatically get their permutation-invariance as well:

**Theorem** **3.** *Let ${\left(X_{n}\right)}_{n\geq 1}$ be a uniformly bounded sequence of random variables with ${∥X_{n}∥}_{2}=1$(n=1,2,…). Then, there exists a subsequence ${\left(X_{n_{k}}\right)}_{k\geq 1}$ and bounded random variables X and Y≥0 such that*

(7) $$\lim_{N\to \infty }A_{N}^{-1}\sum_{k=1}^{N}a_{k}\left(X_{n_{k}}-X\right)\overset{d}{⟶}N\left(0,Y\right)$$

*for any positive numerical sequence ${\left(a_{n}\right)}_{n\geq 1}$ satisfying*

$$A_{N}={\left(\sum_{k=1}^{N}a_{k}^{2}\right)}^{1/2}⟶\infty ,\;\;\max_{k\leq N}\left|a_{k}\right|=o\left(A_{N}\right).$$

*Moreover, relation (7) remains valid after any permutation of the sequence ${\left(X_{n_{k}}\right)}_{k\geq 1}$.*

**Theorem** **4.** *Let ${\left(X_{n}\right)}_{n\geq 1}$ be a uniformly bounded sequence of random variables with ${∥X_{n}∥}_{2}=1$(n=1,2,…). Then, there exist a subsequence ${\left(X_{n_{k}}\right)}_{k\geq 1}$ and bounded random variables X and Y≥0 such that (5) holds for any positive numerical sequence ${\left(a_{n}\right)}_{n\geq 1}$ satisfying (6). Moreover, relation (5) remains valid after any permutation of the sequence ${\left(X_{n_{k}}\right)}_{k\geq 1}.$*

The unpermuted forms of Theorems 3 and 4 are due to Gaposhkin [17]. To deduce the permutation-invariant forms from Theorem 1, it suffices to use the version of the CLT and LIL for independent random variables due to Kolmogorov [18] and Lévy ([19], p. 105), the permutation-invariance of conditional independence and the observation that by (4) we have

$$\sum_{k=1}^{N}|a_{k}\left|\right|X_{n_{k}}-Y_{k}|\leq (\max_{k\leq N}|a_{k}\left|\right)\sum_{k=1}^{N}\left|X_{n_{k}}-Y_{k}\right|=o\left(A_{N}\right).$$

Next, we formulate a version of the Kolmogorov–Erdos–Feller–Petrowski upper–lower class test for lacunary series.

**Theorem** **5.** *Let ${\left(X_{n}\right)}_{n\geq 1}$ be a uniformly bounded sequence of random variables with ${∥X_{n}∥}_{2}=1$(n=1,2,…). Then there exists a subsequence ${\left(X_{n_{k}}\right)}_{k\geq 1}$ and bounded random variables X and Y≥0 such that for any numerical sequence ${\left(a_{n}\right)}_{n\geq 1}$ satisfying*

(8) $$A_{N}={\left(\sum_{k=1}^{N}a_{k}^{2}\right)}^{1/2}⟶\infty ,\;\;\max_{k\leq N}\left|a_{k}\right|=o\left(\frac{A_{N}}{{\left(\log\log A_{n}\right)}^{3/2}}\right)$$

*we have*

(9) $$P\left(\sum_{k=1}^{n}a_{k}X_{n_{k}}>Y^{1/2}A_{n}\varphi \left(A_{n}\right)\;i.o.\right)=1\;\mathrm{or}\;0$$

*according as*

(10) $$\sum_{n=1}^{\infty }\frac{\varphi (n)}{n}e^{-\varphi {\left(n\right)}^{2}/2}=\infty \;\mathrm{or}\;<\infty .$$

*Moreover, the result remains valid after any permutation of the sequence ${\left(X_{n_{k}}\right)}_{k\geq 1}$.*

Again, the result follows from the corresponding result for independent random variables in [20], Theorem 1, and the observation that

$$\sum_{k=1}^{N}|a_{k}\left|\right|X_{n_{k}}-Y_{k}|\leq (\max_{k\leq N}|a_{k}\left|\right)\sum_{k=1}^{N}\left|X_{n_{k}}-Y_{k}\right|=o\left(A_{N}/{\left(\log\log A_{N}\right)}^{3/2}\right).$$

Replacing the exponent 3/2 in (8) by 1/2<ρ<3/2, the upper–lower class test (9)–(10) will continue to hold with new terms appearing gradually in the exponent of $e^{-\varphi {\left(n\right)}^{2}/2}$ as ρ approaches 1/2, according to the hierarchy of results described in [20].

In conclusion, we formulate a weighted strong law for lacunary sequences.

**Theorem** **6.** *Let ${\left(X_{n}\right)}_{n\geq 1}$ be a sequence of random variables, let p,q>1 satisfying 1/p+1/q=1, and assume that there exists a random variable $X\in L_{p}$ such that*

(11) $$P(|X_{n}\left|>t\right)\leq CP\left(|X|>t\right)\;\;\mathrm{for}\;\mathrm{some}\;\mathrm{constant}\;C>0\;\mathrm{and}\;n=1,2,\ldots .$$

*Then there exist a subsequence ${\left(X_{n_{k}}\right)}_{k\geq 1}$ and a random variable $X\in L_{p}$ such that for any array ${\left(a_{N,i}\right)}_{N\geq 1,1\leq i\leq N}$ satisfying*

(12) $$\underset{N}{\sup}{\left(\frac{1}{N}\sum_{i=1}^{N}{\left|a_{N,i}\right|}^{q}\right)}^{1/q}<\infty$$

*we have*

$$\lim_{N\to \infty }\frac{1}{N}\sum_{i=1}^{N}a_{N,i}\left(X_{n_{i}}-X\right)=0\;\;a.s.$$

*Moreover, the result remains valid after any permutation of the sequence ${\left(X_{n_{k}}\right)}_{k\geq 1}$.*

This follows immediately from Theorem 1 and the conditional version of Theorem 1.1 of [21] upon observing that (4) and (12) imply

$$\frac{1}{N}\sum_{i=1}^{N}a_{N,i}\left|X_{n_{i}}-Y_{i}\right|\leq {\left(\frac{1}{N}\sum_{i=1}^{N}{\left|a_{N,i}\right|}^{q}\right)}^{1/q}{\left(\sum_{i=1}^{N}{\left|X_{n_{i}}-Y_{i}\right|}^{p}\right)}^{1/p}N^{-(q-1)/q}$$

and noting that the identical distribution of r.v.’s in Theorem 1.1 of [21] can be replaced by the stochastic domination condition (11). Finally, the permutation-invariance statement follows from the permutation-invariance of conditional independence.

Note that the the assumption (11) and $X\in L_{p}$ made on $X_{n}$ in Theorem 6 are stronger than the moment boundedness condition $\sup_{n}E{\left|X_{n}\right|}^{p}<\infty$, typically assumed in the theory of lacunary series. Whether Theorem 6 remains valid under $\sup_{n}E{\left|X_{n}\right|}^{p}<\infty$ remains open.

In conclusion we mention an interesting recent paper of Karatzas and Schachermayer [22] on the weak law of large numbers for lacunary series. The main result of [22] can also be deduced by using Theorem 1 and the classical criteria for the weak law of large numbers for independent random variables, but since such a proof would not be simpler than the original proof in [22], we do not discuss this problem here.

## Data Availability

Data is contained within the article.
